# Enzyme–adenylate structure of a bacterial ATP-dependent DNA ligase with a minimized DNA-binding surface

**DOI:** 10.1107/S1399004714021099

**Published:** 2014-10-29

**Authors:** Adele Williamson, Ulli Rothweiler, Hanna-Kirsti Schrøder Leiros

**Affiliations:** a Department of Chemistry, UiT The Arctic University of Norway, N-9037 Tromsø, Norway; b NorStruct, Department of Chemistry, UiT The Arctic University of Norway, N-9037 Tromsø, Norway

**Keywords:** ATP-dependent DNA ligase, *Psychromonas* sp. strain SP041

## Abstract

DNA ligases are a structurally diverse class of enzymes which share a common catalytic core and seal breaks in the phosphodiester backbone of double-stranded DNA *via* an adenylated intermediate. Here, the structure and activity of a recombinantly produced ATP-dependent DNA ligase from the bacterium *Psychromonas* sp. strain SP041 is described. This minimal-type ligase, like its close homologues, is able to ligate singly nicked double-stranded DNA with high efficiency and to join cohesive-ended and blunt-ended substrates to a more limited extent. The 1.65 Å resolution crystal structure of the enzyme–adenylate complex reveals no unstructured loops or segments, and suggests that this enzyme binds the DNA without requiring full encirclement of the DNA duplex. This is in contrast to previously characterized minimal DNA ligases from viruses, which use flexible loop regions for DNA interaction. The *Psychromonas* sp. enzyme is the first structure available for the minimal type of bacterial DNA ligases and is the smallest DNA ligase to be crystallized to date.

## Abstract 2

The enzyme–adenylate structure of a bacterial ATP-dependent DNA ligase (ADL), which does not have any additional DNA-binding domains, is similar to minimal viral ADLs that comprise only the core catalytic domains. The bacterial ADL also lacks the unstructured loops which are involved in DNA binding in the viral ADLs, implying that it must instead use short well structured motifs of the core domains to engage its substrate.

## 1. Introduction   

The sealing of breaks in the phosphodiester backbone of double-stranded DNA is essential for the replication and survival of all organisms. This function is carried out by DNA ligases in a three-step reaction in which the enzyme first self-adenylates, followed by DNA binding and transfer of AMP to the 5′-phosphate terminus of the DNA nick, which is then activated for nucleophilic attack by the apposing 3′-OH group. This final step creates a new phosphodiester bond and releases the AMP.

DNA ligases are members of the nucleotidyltransferase superfamily and are similar to the GTP-dependent mRNA-capping enzymes (Shuman & Lima, 2004 ▶). The nature of the AMP donor for DNA ligases divides them into two classes: the highly conserved NAD-dependent enzymes, which are found in all bacteria and carry out the essential joining of Okazaki fragments during DNA replication (Wilkinson *et al.*, 2001 ▶), and the structurally diverse ATP-dependent enzymes, which are found in all domains of life (Martin & MacNeill, 2002 ▶). This division in cofactor preference is reflected in distinct structural differences, with NAD-dependent DNA ligases possessing a unique N-terminal Ia domain which is essential for utilization of this substrate (Sriskanda & Shuman, 2002 ▶). ATP-dependent DNA ligases (abbreviated ADLs) share a common catalytic core which includes all six of the conserved nucleotidyltransferase motifs and comprises the adenylation domain (AD domain), where the AMP cofactor is covalently bound, and an oligonucleotide-binding domain (OB domain), which engages the minor groove of the DNA duplex upon nick binding and assists in the step 1 adenylation reaction (Shuman, 2009 ▶; Doherty & Suh, 2000 ▶). These two essential domains are appended with a variety of organism-specific domains and motifs which are involved in DNA binding (Pascal *et al.*, 2004 ▶; Nair *et al.*, 2007 ▶), cellular localization (Lakshmipathy & Campbell, 1999 ▶) or the recruitment of other protein inter­action partners (Pascal *et al.*, 2006 ▶; Liu *et al.*, 2013 ▶; Kiyonari *et al.*, 2006 ▶), or in some cases have independent enzymatic functions (Zhu *et al.*, 2006 ▶; Zhu & Shuman, 2005 ▶, 2006 ▶).

Enzyme–substrate intermediates in the ligation reaction have been crystallized for a number of ADLs, including several ligase–adenylate structures (Kiyonari *et al.*, 2006 ▶; Subramanya *et al.*, 1996 ▶; Odell *et al.*, 2000 ▶; Nishida *et al.*, 2005 ▶; Akey *et al.*, 2006 ▶) and three with nicked DNA bound in the active site: the small *Chlorella* virus PBCV-1 DNA ligase (hereafter referred to as ChlV-Lig; Nair *et al.*, 2007 ▶) and the two large eukaryotic human DNA ligases I and III (Pascal *et al.*, 2004 ▶; Cotner-Gohara *et al.*, 2010 ▶). Comparison of the DNA-bound and DNA-free forms of these proteins indicate that their substrate-binding mode is mechanistically conserved, with the enzyme fully encircling the DNA strand, but that different protein structures are used to accomplish this. In both cases the protein transitions from an open conformation in which the DNA-binding sites of the AD and OB domains are oriented away from each other to a closed conformation by rotation of the two domains about their flexible linker section (Shuman, 2009 ▶). In ChlV-Lig the ring structure around the DNA is completed by ordering of a large loop region that protrudes from the OB domain and wraps around the strand to form contacts with the AD domain, while in ligases I and III encirclement involves a large N-terminal DNA-binding domain comprising 12 α-helices.

All bacterial genomes encode an essential NAD-dependent DNA ligase which joins Okazaki fragments during DNA replication, but some species also have additional ATP-dependent proteins (Wilkinson *et al.*, 2001 ▶). Bacterial ADLs appear to fall into two broad categories: large intracellular enzymes that are involved in double-stranded break repair (Pitcher *et al.*, 2007 ▶) and smaller proteins, many of which possess a predicted periplasmic leader peptide. The first group, typified by the LigD ADLs from *Bacillus subtillis* and *Mycobacterium tuberculosis*, require the DNA end-binding protein Ku for optimal activity (Weller *et al.*, 2002 ▶; Della *et al.*, 2004 ▶) and in many cases are multi-functional with additional DNA-repair activities imparted by enzymatic domains in the same polypeptide chain (Zhu & Shuman, 2005 ▶, 2006 ▶; Zhu *et al.*, 2005 ▶, 2006 ▶, 2012 ▶; Wright *et al.*, 2010 ▶). To date, the only structure of a bacterial ADL that has been determined is that of the large multi-functional *M. tuberculosis* LigD protein (hereafter referred to as Mtu-Lig), the C-terminal ligase domain of which (residues 452–759) was crystallized in an adenylated form (Akey *et al.*, 2006 ▶), while the X-ray structure of the primase/polymerase domain was determined separately (Zhu *et al.*, 2006 ▶). The second type of bacterial ADLs, which are found predominantly in the Proteobacteria, only have the minimal core architecture and show high rates of intrinsic nick-sealing activity (Cheng & Shuman, 1997 ▶; Magnet & Blanchard, 2004 ▶). Two such ‘minimal’ ADLs from the human pathogens *Neisseria meningitidis* (hereafter referred to as Nme-Lig; Magnet & Blanchard, 2004 ▶) and *Haemophilus influenzae* (hereafter referred to as Hin-Lig; Cheng & Shuman, 1997 ▶) have been recombinantly produced and their enzymatic activities have been extensively characterized. Both are capable of sealing single-nicked double-stranded DNA (ds-DNA) without requiring additional protein-interaction partners and both have relatively high affinity for both ATP and DNA substrates. Recently, preliminary characterization of recombinant ADL from the psychrophilic fish pathogen *Aliivibrio salmonicida* (hereafter referred to as Vib-Lig) showed increased rates of nick sealing when the protein did not include the N-terminal leader sequence, which provides strong support for the periplasmic location of these enzymes in the native host (Williamson & Pedersen, 2014 ▶).

Here, we report the biochemical and structural characterization of a bacterial ADL present in the partially sequenced genome of the psychrotolerant *Psychromonas* sp. strain SP041 isolated from Svalbard, Norway. This protein, hereafter referred to as Psy-Lig, is a minimal type of bacterial ADL possessing only the catalytic domains necessary for self-adenylation and nick-sealing and an N-terminal periplasmic localization sequence. The 1.65 Å resolution structure of the enzyme–adenylate complex presented here represents only the second structure of a bacterial ADL to date and is the only available structure of the minimal type of bacterial ADL. At 257 residues, it is also the smallest DNA ligase that has been structurally studied, being 41 residues shorter than the minimal ChlV-Lig protein, and comparison with this homologue suggests that it may use an alternative mode of engaging its DNA substrate to those previously described.

## 2. Experimental procedures   

### 2.1. Cloning and expression of Psy-Lig   

Psy-Lig was identified at positions 201749–202642 (reverse strand) in the genome of *Psychromonas* sp. strain SP041 (GenBank accession No. ERP003516) using *Glimmer*3 for gene prediction (Delcher *et al.*, 2007 ▶) and homology searches against the nonredundant protein sequences database. *SignalP* 4.1 (Gram-negatives networks setting, *D* cutoff value 4.2; Petersen *et al.*, 2011 ▶) predicted a periplasmic targeting leader sequence of 40 amino acids with a cleavage position between residues Ala40 and Gln41. A codon-optimized synthetic DNA construct (Eurofins) encoding the mature protein along with an N-terminal His tag and TEV protease site was inserted into the entry vector pDONR22 and then subsequently cloned into and expressed from the Gateway destination vector PHMGWA (GenBank number EU680841). All cloning, expression and purification steps were carried out as described previously for the homologous protein from *A. salmonicida* (Williamson & Pedersen, 2014 ▶). The clarified lysate was incubated overnight with 0.1 m*M* ATP to ensure complete adenylation of the purified enzyme.

Production of SeMet-substituted Psy-Lig was performed by cultivation of *Escherichia coli* BL21(DE3)Star PlysS/PHMGWA::*psy-Lig* cells in M9 minimal medium at 37°C until an OD_600_ of 0.4 was reached, whereupon supplementary amino acids were added at the following concentrations: l-lysine, l-phenylalanine and l-threonine at 100 mg l^−1^, l-isoleucine, l-leucine and l-valine at 50 mg l^−1^ and l-selenomethinone at 60 mg l^−1^. Cultivation was continued for 1 h at 37°C and the temperature was then decreased to 22°C. After 30 min of equilibration time, protein production was induced by the addition of 1 m*M* IPTG and expression was allowed to proceed overnight. SeMet-substituted Psy-Lig was purified as described for the native protein and the extent of selenium incorporation was determined by electrospray ionization mass spectrometry of the labelled and native proteins. Enzymatic activity of the SeMet-substituted preparation was verified using the real-time assay described below.

### 2.2. Crystallization and structure determination   

#### 2.2.1. Crystallization and data collection   

The SeMet incorporation was estimated to be 95% by mass spectrometry and the substituted enzyme was active in nick-sealing, with similar rates to those of the native protein (data not shown). Prior to the commencement of crystallization trials, fresh SeMet-substituted Psy-Lig protein was concentrated to 8.0 mg ml^−1^. The protein was crystallized using the hanging-drop vapour-diffusion method at 4°C from 0.1 *M* HEPES pH 7.4, 1.6–1.9 *M* ammonium sulfate as a precipitant. Crystals appeared after 2 days and reached maximum size in approximately one week. All crystals were cryoprotected using 1.4 *M* ammonium sulfate, 0.1 *M* HEPES pH 7.5, 27% ethylene glycol and were flash-cooled in liquid nitrogen. Data were collected at the BESSY II synchrotron, Berlin, Germany.

To obtain the phases, a MAD experiment was performed on SeMet-labelled Psy-Lig crystallized from 1.9 *M* ammonium sulfate. Anomalous data were collected to 2.9 Å resolution based on a fluorescence scan of the selenium at peak, inflection and remote energies (12.6545, 12.6525 and 12.6845 keV, respectively). A subsequent high-resolution data set was collected from a second SeMet crystal crystallized using 1.75 *M* ammonium sulfate, which diffracted to 1.65 Å resolution (Table 1 ▶).

#### 2.2.2. Structure determination and refinement   

**Figure 1:**
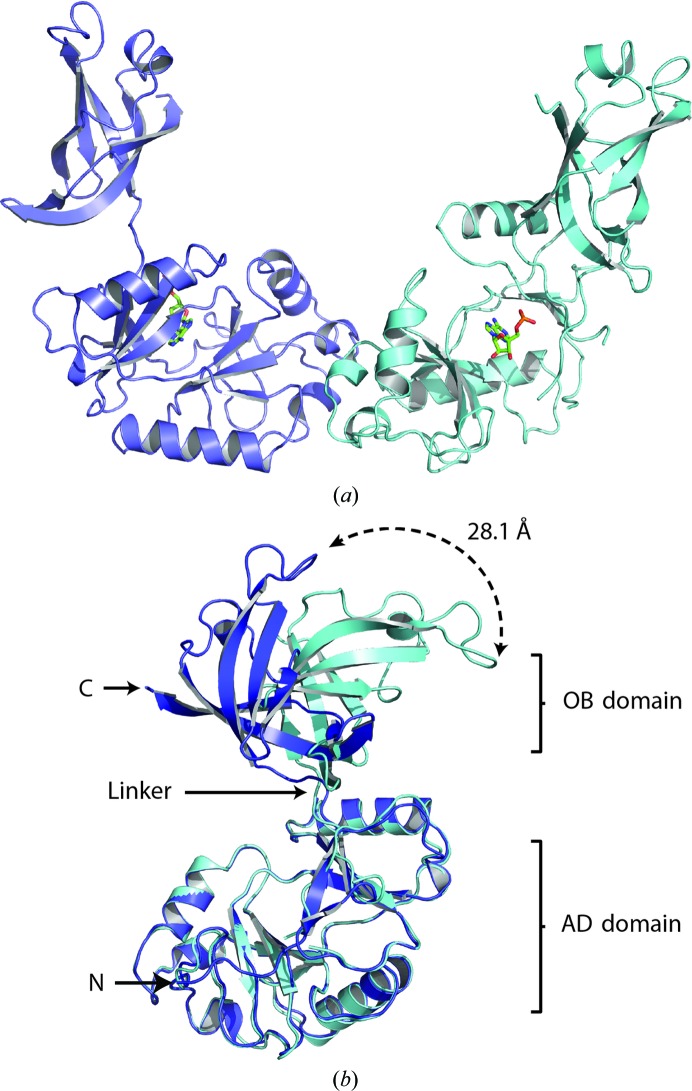
Overall fold of Psy-Lig chain *A* (purple) and chain *B* (blue). The covalently bound AMP is shown in green. (*a*) Chains in the asymmetric unit. (*b*) Superposition of chains by alignment of their AD domains. The dashed arrow indicates the distance between the C^α^ atom of Gly191 of each monomer.

A substructure of 13 Se sites was found in *SHELXD* with occupancies of 1.0–0.5 using the three-wavelength MAD data set to 2.9 Å resolution. This was followed by phase extension and phase modification in *SHELXE* (Sheldrick, 2010 ▶) using the high-resolution SeMet data to 1.65 Å resolution, which allowed *ARP*/*wARP* (Langer *et al.*, 2008 ▶) to build a partial model of 490 residues. Subsequent rounds of manual model building in *WinCoot* (Emsley & Cowtan, 2004 ▶) and refinement in *PHENIX* (Afonine *et al.*, 2012 ▶) gave the final model, which was deposited in the Protein Data Bank as entry 4d05 (Table 1 ▶). Psy-Lig crystallized in space group *C*2 with two monomers in the asymmetric unit (Fig. 1 ▶ *a*).

### 2.3. Enzyme assays   

#### 2.3.1. DNA substrates   

To study the DNA ligase activity of Psy-Lig, two different assays were used: a TBE–urea gel-based endpoint assay to investigate activity on DNA substrates with single, double, gapped or mismatched nicks and a time-resolved assay using a molecular beacon (MB) to measure the rate of single nick sealing (Tang *et al.*, 2003 ▶). To allow a good comparison between the two methods, the same nucleotide (nt) sequence was used as a basis for all oligo­nucleotide substrates in both assays (Table 2 ▶). In the case of the endpoint TBE–urea gel assay the 5′ end of the nicked strand was labelled with 5-carboxyfluorescein (FAM), while in the real-time MB assay the fluorescent reporter moiety tetramethyl­rhodamine (TAMRA) was carried on the complementary strand and was quenched by the adjacent 4-(4′-dimethyl­aminophenylazo)benzoic acid (DABCYL) molecule, which was held in close proximity in the nicked state as described by Tang *et al.* (2003 ▶). HPLC-purified oligomers (Sigma) were used without any further purification. The ratio of oligo­nucleotides used to form the DNA duplex was 4:3 complement to nick strands (Table 3 ▶). In all cases ‘substrate concentration’ refers to the concentration of labelled nick strands in the reaction.

#### 2.3.2. Gel-based assays   

Gel-based endpoint assays were carried out as described previously (Magnet & Blanchard, 2004 ▶; Cheng & Shuman, 1997 ▶) with the modification that the 5′-fluorophore FAM was substituted for the 5′-^32^P label (Wang *et al.*, 2013 ▶). Reaction volumes were 10 µl, unless otherwise specified, and consisted of 2.0 n*M* enzyme, 80 n*M* substrate, 0.1 m*M* ATP, 10 m*M* MgCl_2_, 1 m*M* DTT, 100 m*M* NaCl and 50 m*M* Tris–HCl pH 8.0. Enzymatic activity was detected by conversion of the FAM-labelled 9 nt substrate oligonucleotide into an 18 nt product after sealing of the nick in the double-stranded substrate. The bands were resolved by denaturing electrophoresis on a 20% acrylamide/7 *M* urea/1× Tris–borate–EDTA (TBE) gel, detected by fluorescence on a Pharos FX Plus imager (Bio-Rad) and quantified by band intensity using the *ImageJ* software (Schneider *et al.*, 2012 ▶). The extent of ligation activity was taken as the ratio between the upper 18 nt product band and the lower 9 nt substrate band and was expressed as a percentage. Enzyme activity with the different DNA complexes was tested at 15°C for either 5 min (nicked, cohesive and mismatch substrates), 30 min (gapped substrate) or over a 25 h time course (blunt, non­phos­phorylated and single-stranded substrates) using the enzyme concentrations specified in the figure captions. The optimal NaCl concentration for activity was tested at 30°C for 5 min.

#### 2.3.3. Molecular beacon assay   

A time-resolved molecular beacon (MB) assay was carried out as previously described (Tang *et al.*, 2003 ▶). Unless otherwise stated, the reaction conditions were 2.0 n*M* Psy-Lig, 300 n*M* substrate, 0.1 m*M* ATP, 10 m*M* MgCl_2_, 1 m*M* DTT, 100 m*M* NaCl, 50 m*M* Tris pH 8.0. Reactions were carried out in a 96-well plate (Corning, black flat-bottom low protein-binding surface) in a volume of 100 µl. Fluorescence was measured using a Spectromax M2^e^ (Molecular Devices) with an excitation wavelength of 521 nm and an emission wavelength of 578 nm. The assay setup was allowed to equilibrate at 30°C for at least 30 min before initiation by the addition of enzyme. Reactions were mixed for 5 s subsequent to enzyme addition and the fluorescence emission was recorded every 5 s for 30 min. The kinetics of nick sealing by Psy-Lig were investigated by varying the concentration of either ds-DNA or ATP while the other substrate was kept constant and in excess. Steady-state kinetics were measured for concentrations of the MB between 11.25 and 300 n*M* in the presence of 0.1 m*M* ATP and 1.0 n*M* enzyme; however, for the reasons described in §[Sec sec3]3 only the concentration range between 11.25 and 225 n*M* was used for the calculation of *K*_m_ and *k*_cat_. ATP kinetics were measured at ATP concentrations between 0 and 50 µ*M* with 300 n*M* MB substrate and 2.0 n*M* Psy-lig. To quantify the fluorescence change resulting from nick sealing, calibration curves were constructed by serial dilution of the singly nicked MB substrate and the fully ligated substrate where the two short oligomers were replaced with an 18 nt strand as described in Tang *et al.*, 2003 ▶). The conversion value used in calculations was the difference between these as the nicked substrate had significant background fluorescence. Kinetic rates were calculated from the initial slope of the progress curves and were fitted to the Michaelis–Menten equation using the kinetics module of *SigmaPlot*.

## 3. Results   

### 3.1. Crystal structure of Psy-Lig   

#### 3.1.1. Overall structure   

Each Psy-Lig monomer has a large (172-residue) N-terminal AD domain comprising two four-stranded β-sheets surrounded by five α-helices and a smaller (77-residue) OB domain comprising a five-stranded β-barrel and a single α-helix. All backbone and side chains are resolved for chain *A* (Psy-Lig-*A*), but in chain *B* (Psy-Lig-*B*) eight residues had no electron density and were left out of the model: four in the N-terminus, two in the C-terminus and two in the loop between strands β7 and β8. The two domains are connected by an eight-residue linker, and in both Psy-Lig chains these adopted the open conformation typical of the enzyme–adenylate intermediate, in which the DNA-binding surfaces of the two domains face away from each other (Nair *et al.*, 2007 ▶; Tomkinson *et al.*, 2006 ▶; Shuman, 2009 ▶). The two chains in Psy-Lig have a C^α^ r.m.s.d. difference of 3.45 Å; however, when they are structurally aligned based only on the AD domains it is clear that this arises primarily from a different orientation of their OB domains about the linker (Fig. 1 ▶ *b*). When the AD domains are aligned, the measured distance of the C^α^ atom of Gly191 in the OB domain of each chain is 28.1 Å. If the AD and OB domains are aligned separately, the r.m.s.d. values are only 0.63 and 0.72 Å, respectively. The more complete electron density of Psy-Lig-*A* is reflected by its lower *B* factor (11.6 Å^2^) compared with chain *B* (32.8 Å^2^). For this reason, it is chain *A* which is referred to in the subsequent sections of the discussion unless otherwise specified.

**Figure 2:**
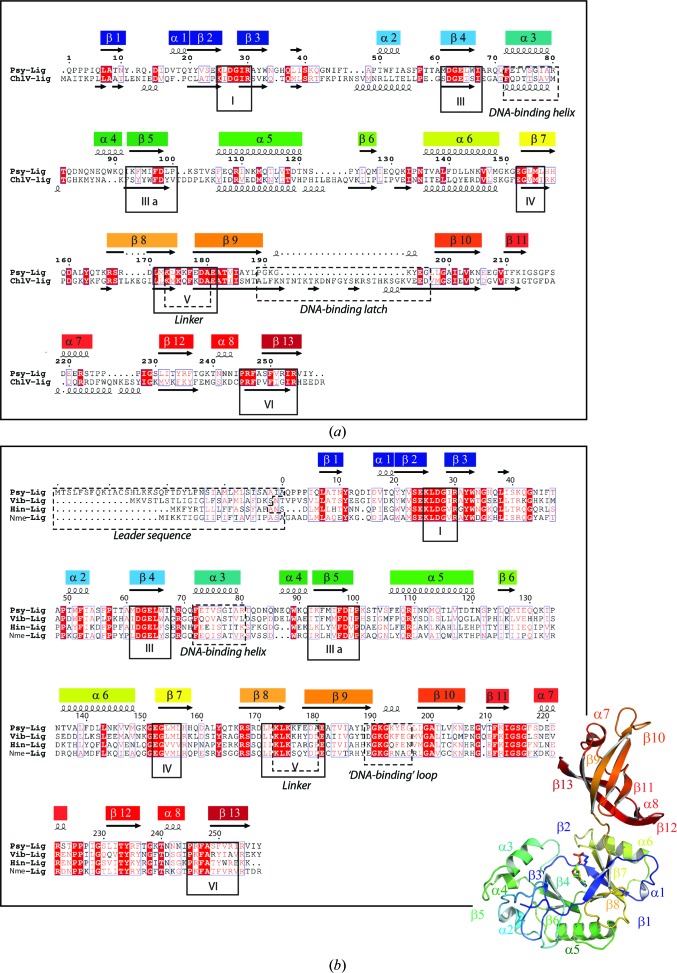
Sequence alignments of Psy-Lig with other ATP-dependent DNA ligases. Fully conserved residues are shaded red; partially or homologously conserved residues are shown in red text. Structural features discussed in the text and the conserved motifs of the nucleotidyltransferase enzymes are indicated in boxes, with numbering corresponding to the conventions given in Shuman & Lima (2004 ▶). α-Helices are indicated by curl symbols and β-strands by arrows. Filled boxes above the sequences give the numbering of the secondary-structural elements of Psy-Lig as referred to in the text, and correspond to the colours and numbering used in the ribbon diagram (inset). (*a*) Structure-based alignment between Psy-Lig and ChlV-­Lig constructed using *PDBeFold*. (*b*) Sequence-based alignment of Psy-Lig with biochemically characterized ligases from *H. influenzae* (Hin-Lig), *N. meningitidis* (Nme-Lig) and *A. salmonicida* (Vib-­Lig).

A structure-based homology search using the *PDBeFold* server found the closest matches to the overall Psy-Lig structure were the adenylated and DNA-bound structures of the *Chlorella virus* ATP-dependent DNA ligase (abbreviated ChlV-Lig–AMP and ChlV-Lig–DNA, respectively; PDB entries 1fvi and 2q2t; Nair *et al.*, 2007 ▶; Odell *et al.*, 2000 ▶). Less significant matches were also found to ATP-dependent DNA ligases from the T7 phage (T7-Lig; PDB entry 1a0i; Subramanya *et al.*, 1996 ▶) and the ligase domain of *M. tuberculosis* LigD (Mtu-Lig; PDB entry 1vs0; Akey *et al.*, 2006 ▶) (Table 4 ▶). Separate searches for Psy-Lig using the only the AD (residues 1–176) or OB domains (residues 177–257) revealed a significant difference in structure between the OB domains from Psy-Lig and Mtu-Lig. While the AD domain is very similar in all five structures, the OB domain of Psy-Lig more closely resembles those of the two viral proteins rather than the other bacterial homologue. These structural differences are reflected by the extremely divergent primary sequences between the two bacterial proteins, which have almost no significant similarity at the amino-acid level (Table 4 ▶). A structure-based sequence alignment between Psy-Lig and ChlV-Lig found 27% identity (56 residues) and 30% similarity (62 residues; Fig. 2 ▶ *a*). All of the conserved motifs of the nucleotidyltransferase family are present in Psy-Lig, as is the α3 helix which was shown to interact with the minor groove of the 3′ side of the nicked strand of the DNA in the ChlV-Lig–DNA structure (Nair *et al.*, 2007 ▶). The most significant differences are the truncation of the OB-domain β9–β10 loop and a shorter α7 helix in Psy-Lig relative to ChlV-Lig, which are discussed in more detail below.

#### 3.1.2. Enzyme adenylate and active site   

**Figure 3:**
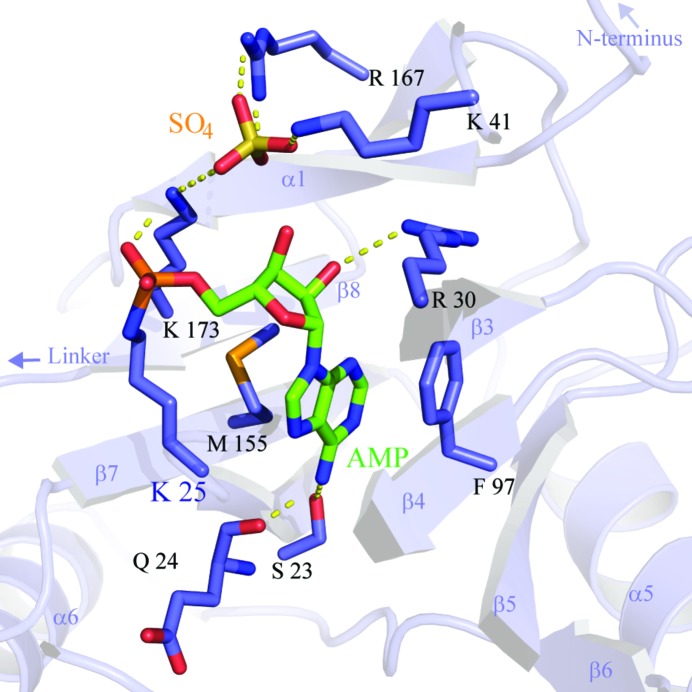
Active-site structure of Psy-Lig-*A*. The nucleotide cofactor is depicted and labelled in green and the sulfate ion is in yellow. Lys25 which is covalently attached to the AMP is labelled in bold; all residues making contacts with the cofactor and ion are shown as purple sticks. Secondary-structural elements of the AD domain are labelled in light purple and correspond to the notation used in Fig. 2 ▶.

AMP bound in the active site was clear in the observed electron density, covalently linked to Lys25 *via* a phosphoamide bond to the side-chain N^ζ^ atom, which is consistent with exposure of the protein to high levels of ATP during purification (Fig. 3 ▶). Nine sulfate molecules were also resolved in the electron density, one of which is located in the active site of chain *A*. A sulfate ion at the equivalent position in ChlV-Lig–AMP occupies the binding site of the nicked 5′ DNA in ChlV-Lig–DNA, a structure which is also likely to be the case here. As in other ligase–adenylate structures, the AMP is in the *anti* conformation, with the adenine nucleotide base stacked between the hydrophobic side chains of Phe97 in motif IIIa and Met155 in motif IV. The side chains of Ser32 and Glu24 provide hydrogen bonds to the exocyclic N6 of the base, while the side chain of Arg30 in motif I is positioned to hydrogen bond to the ribose O2. Contacts to the sulfate are made by Lys41 in the β3–α2 loop and Arg167 in β8. Lys173 of motif V also makes a bifurcated bond to the sulfate and the nonbridging phosphate O atom of the adenylate (Fig. 3 ▶).

#### 3.1.3. Domain dynamics and modelling of DNA inter­action   

**Figure 4:**
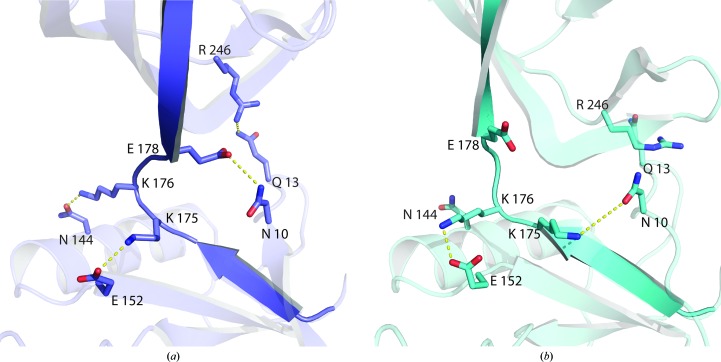
Conformation of the linker between the AD and OB domains. (*a*) Chain *A*, (*b*) chain *B*.

**Figure 5:**
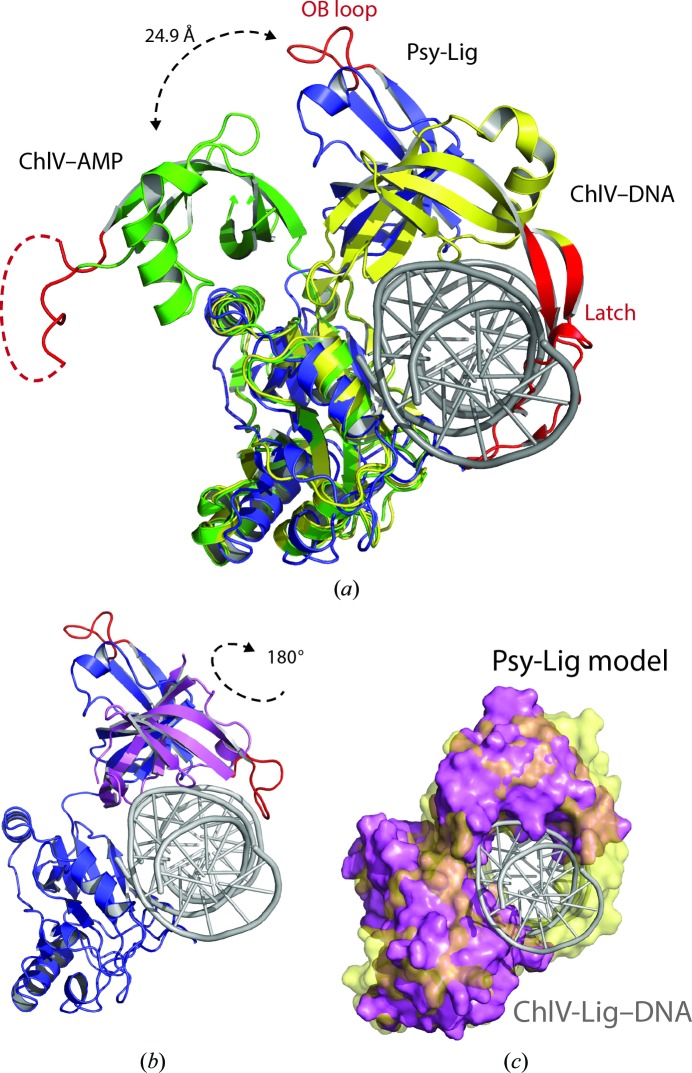
Comparison of the tertiary structure of Psy-Lig with that of ChlV-Lig. (*a*) Superposition of Psy-Lig chain *A* (purple) with ChlV-Lig–AMP (green, PDB entry 1fvi) and ChlV-Lig–DNA (yellow, PDB entry 2q2t). The DNA-binding latch of ChlV-Lig and the homologous loop of Psy-Lig are coloured red. The unstructured residues of ChlV-Lig–AMP are indicated as a dashed line. DNA is shown in grey. (*b*) Psy-Lig modelled in the DNA-bound conformation by superposition of the Psy-Lig OB domain with the ChlV-Lig–DNA OB domain–DNA complex. The original adenylated Psy-Lig structure (purple) and the re-oriented position (pink) are shown as ribbons. The DNA from the ChlV-Lig–DNA structure is shown in grey. (*c*) Surface of the Psy-Lig DNA-bound model (pink) superposed with the ChlV-Lig–DNA structure (yellow, transparent), showing incomplete encirclement of the DNA by Psy-Lig.

Interactions between the AD and OB domains of Psy-Lig-*A* include hydrogen bonds between Arg246 (loop α8–β13) and Gln13 (loop β1–α1), and between Gly238 (loop β12–α8) and Asn10 (β1) *via* hydrogen bonding to the side chain of the linker residue Glu178. The AD domain also forms a salt bridge to the linker from Glu152 of β7 to Lys175 and a hydrogen bond from Asn144 of α6 to Lys176 (Fig. 4 ▶ *a*). In Psy-Lig-*B* the OB domain is tilted further away from the AD domain, which equates to a one-residue turn in the linker–AD hydrogen-bonding pattern. Instead, Glu152 forms an ion pair with Lys176 rather than Lys175, which has no intramolecular hydrogen bonds (Fig. 4 ▶ *b*). Interactions between Glu178 and Asn10 and between Arg246 and Gln13 are not present in Psy-Lig-*B*. Superposition of the structures of Psy-Lig-*A*, ChlV-Lig–AMP and ChlV-Lig–DNA using only their AD domains for alignment shows that the OB domain of Psy-Lig-*A* occupies an intermediate position between those of the DNA-bound (open) and DNA-free (closed) forms of ChlV-Lig (Fig. 5 ▶ *a*). The top of α7 is tilted around 25 Å more towards the DNA-bound position than ChlV-Lig–AMP, but the OB domain would still need to swivel about the linker to be oriented the same as in ChlV-Lig–DNA. The position of the OB domain of Psy-Lig-*B* is closer to that of ChlV-Lig–AMP. To gain insight into its possible DNA-binding interactions, the DNA-bound orientation of Psy-Lig was modelled by superimposing the OB domain onto the OB domain of the ChlV-Lig–DNA structure (PDB entry 2q2t; Fig. 5 ▶ *b*). This involved a swivel of 180° about the linker but did not require a translation relative to the AD domain. The DNA from the ChlV-Lig–DNA structure fits well into the concave surface of the Psy-Lig OB domain, with the complementary strand running 3′ to 5′ from the linker to the loop end, while the nicked strand runs 3′ to 5′′ across the flatter AD domain surface. Together, the two Psy-Lig domains encircle just over 180° of the circumference of the DNA duplex, in contrast to the complete encirclement by ChlV-Lig (Fig. 5 ▶ *c*).

#### 3.1.4. Surface topology and electrostatics   

**Figure 6:**
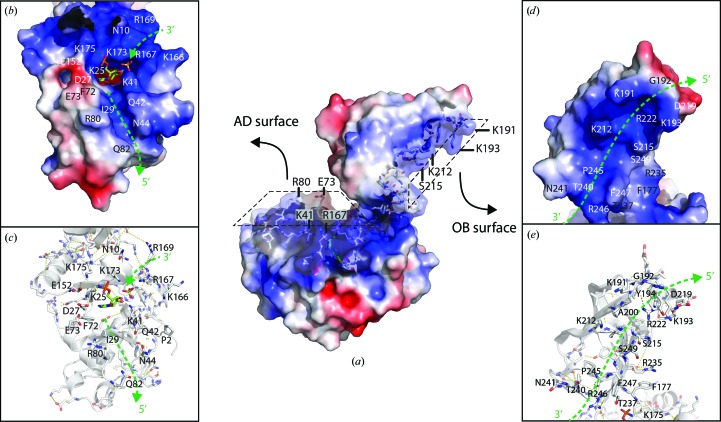
Psy-Lig-*A* coloured by electrostatic surface potential. The surface potential was generated using *APBS* (Dolinsky *et al.*, 2007 ▶), with positively charged areas shown in blue and negatively charged areas in red. (*a*) Overview of the Psy-Lig structure with residues predicted to be involved in DNA interactions shown as sticks. (*b*) Surface and (*c*) important residues of the AD domain looking at the active site. The AMP cofactor and sulfate ion are shown as sticks. Green arrows indicate the approximate positions of the nicked DNA strands based on the model shown in Fig. 5 ▶. (*d*) Surface residues and (*e*) residues of the OB domain looking towards the DNA-binding site predicted by the model. The green arrow indicates the approximate position of the complementary DNA strand in the OB groove.

Analysis of the surface topology and electrostatics of Psy-Lig indicate DNA-interacting residues on both binding faces of the OB and AD domains as well as positive surface potentials that would contribute to DNA binding (Fig. 6 ▶ *a*). The OB domain has a positively charged groove running down its concave surface which could accommodate the unbroken complementary strand of the DNA duplex (Figs. 6 ▶ *c* and 6 ▶ *d*). Partial positive charges in the depth of the groove are provided by the amide N atoms of residues Ile213, Gly214, Ser215 and Gly216 in the loop between β11 and α7, Gly199 and Ala200 in the loop between β9 and the beginning of strand β10, and Ser249, Ala248, Phe247 and Pro245 in the loop between β12 and α8 (the VI motif of nucleotidyltransferases). The sides of the groove are formed by the bulky side chains of residues Lys212, Ser215, Arg222, Asn241, Thr240 and Phe247. The OB-domain loop between β9 and β10 follows the curve of the DNA backbone (Fig. 5 ▶ *b*) and occupies a homologous position to the latch region of ChlV-Lig–DNA. At the tip of the OB-domain loop, a small 3_10_-helix contains two lysine residues, Lys191 and Lys193, which form a rigid fork with their side chains pointing to either side of the groove (Figs. 6 ▶ *c* and 6 ▶ *d*). When the OB domain is oriented in the DNA-binding position, this fork is positioned to straddle the DNA backbone and interact with both the major and minor grooves of the duplex (Fig. 5 ▶ *b*). In Psy-Lig-*A* the side chain of Arg222 (α7) stacks against the side chain of Tyr194, which in turn lies flat against Lys193, forming a continuous structure along one edge of the groove (Figs. 6 ▶ *c* and 6 ▶ *d*). Arg222 also makes two hydrogen bonds (*via* its N1 and O atoms) to the main-chain atoms of Leu198 (at the beginning of β10), one hydrogen bond from N2 to Gly214 (in the β11–α7 loop) and a bifurcated bond from its main-chain N atom to Ser218 and Asp219 (at the N-terminal end of α7). A main-chain interaction between Leu197 and Gly190 pinches across the base of the loop. A second groove on the DNA-interacting face of the AD domain is formed by α3 and residues Lys41, Gln42 and Asn44 of the β3–α2 loop (Fig. 6 ▶ *b*). This would be positioned to fit the 3′ strand of the nicked DNA, and runs across the pocket in whcih the AMP is covalently bound to Lys25. The β3–α2 loop is stabilized by numerous interactions, including main chain to main chain interactions between Ser40 and Gly43, between Ser40 and Asn44 and between Leu38 and Phe46, main chain to side chain inter­actions between His36 and Asn34, between Asn37 and Asn34 and between Asn44 and Ser40, and side chain to side chain interactions between Asn34 and Asn37 and between Ser40 and Asn44. A hydrogen bond between the main chain of Asp83 and the side chain of Thr47 links the two lips of the groove furthest from the AMP site, and an interaction between the side chains of Asp62 and Tyr163 tethers the β3–α2 loop to one of the two β-sheets that form the core of the AD domain. The groove continues to the other side of the active site, where a cluster of positive charges provided by Lys173 and Lys175 of the linker and Asn10 from β1 could provide contacts to the 5′-end of the nicked strand, while Arg169, Arg167 and Lys166 from the β7–β8 loop form a positively charged surface for interaction with the backbone of the complementary strand. Hydrogen bonds between Gln6 and Gln164 and between Ala8 and Arg167 link the N-terminal β1 strand to the β7–β8 loop and position the N-terminus to lie alongside the β3–α2 loop.

### 3.2. Enzyme activity of Psy-Lig   

#### 3.2.1. Ligation of different DNA substrates   

**Figure 7:**
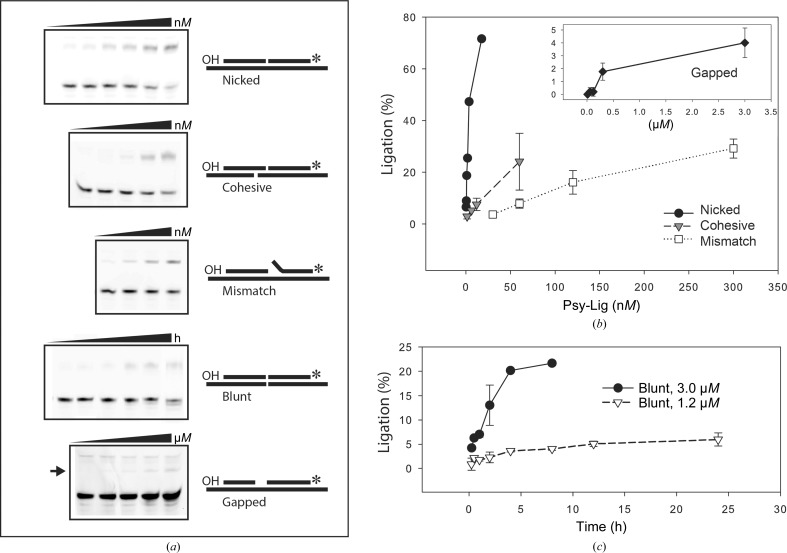
Ligation activity of Psy-Lig with different DNA substrates. (*a*) Schematic representation of DNA substrates and representative results of ligation on TBE–urea gels. The samples for the blunt substrate gel are for a 3.0 µ*M* enzyme concentration over 8 h. The image contrast for the gapped substrate gel has been adjusted to allow visualization of the faint bands from ligation. Integration of bands was carried out prior to this adjustment. (*b*) Percentage of substrate ligated in 5 min for nicked, cohesive and mismatch substrates (main figure) and gapped substrate after 30 min (inset) as a function of enzyme concentration. (*c*) Percentage of blunt substrate ligated over 8 or 24 h for two Psy-Lig dilutions. Ligation activity was quantified by integration of band intensity and is expressed as the percentage of upper (ligated) band relative to the sum of the two bands. Measurements are the mean of three replicate experiments; error bars represent the standard deviation from the mean.

The ligase activity of Psy-Lig on 18 bp DNA duplexes containing centrally placed single-stranded or double-stranded breaks was investigated by a gel-based endpoint assay. Efficient sealing of a perfectly matched 5′-phosphorylated singly nicked substrate in the presence of ATP and MgCl_2_ proceeded within 5 min at 15°C as shown by the appearance of the 18 nt product band on a denaturing TBE–urea gel at the expense of the 9 nt substrate (Fig. 7 ▶ *a*). The extent of nick ligation when quantified by integration of band intensity was dependent on the enzyme concentration, with a linear range between 0.2 and 4.0 n*M* (Fig. 7 ▶ *b*). In addition to sealing a singly nicked fully matched double-stranded DNA substrate, Psy-Lig was also able to ligate a cohesive-ended double-stranded break and a single nick with a 1 bp mismatch at the 3′-OH position of the nick, albeit with reduced efficiency (Fig. 7 ▶ *b*). Ligation of a blunt-ended double-stranded break (Fig. 7 ▶ *c*) and a 1 nt gapped substrate (Fig. 7 ▶ *b*, inset) was also detected when high Psy-Lig concentrations and extended incubation times were used; however, no activity was seen with a nonphosphorylated 5′-nicked strand or single-stranded DNA (data not shown).

#### 3.2.2. Steady-state kinetics and salt optima   

**Figure 8:**
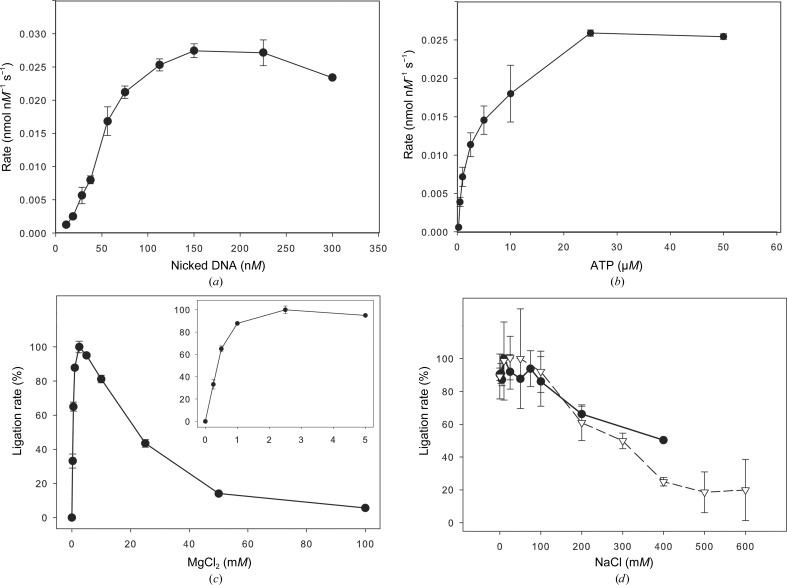
Kinetics of nick sealing by Psy-Lig and the effect of salt concentration measured using the MB assay. (*a*) The DNA (MB substrate) concentration was varied while the ATP concentration was kept at 0.1 µ*M*. (*b*) The ATP concentration was varied while the DNA concentration was kept at 300 n*M*. (*c*) Activity as a function of MgCl_2_ concentration. The inset shows the low-concentration data range for clarity. (*d*) Activity as a function of NaCl concentration measured by the MB assay (solid line, circles) and the endpoint assay (dashed line, triangles). The measurements are the means of three replicate experiments; error bars represent the standard deviation from the mean. The values for the MB assay are the rates over the first 10 min of reaction, while the values for the endpoint assay are the ratio of ligated to unligated substrate. In (*c*) and (*d*) the data were normalized to the maximum rate under that condition and are expressed as a percentage.

The enzyme kinetics of Psy-Lig were measured using the real-time MB assay. As expected, the rate of nick sealing increased with the concentration of the DNA substrate (Fig. 8 ▶ *a*). This increase obeyed the Michaelis–Menten model up to a substrate concentration of 225 n*M* and was used to calculate a *K*_m_ of 124 ± 37 n*M* for nicked DNA and a *k*_cat_ of 0.05 s^−1^. At concentrations of molecular beacon above 300 n*M* the reaction velocity decreased with increasing substrate concentration, and examination of the MB progress curves revealed that this was owing to a delay in the onset of the fluorescence response. TBE–urea gel experiments with substrate concentrations between 25 and 1200 n*M* (5 min at 30°C) showed no evidence of substrate inhibition (data not shown); thus, this behaviour can be attributed to a limitation of the assay, possibly because high concentrations of beacon lead to intermolecular quenching. Reaction rates measured with increasing concentrations of ATP (0–50 µ*M*) showed that ATP is an essential cofactor for DNA ligation, with a *K*_m_ of 3.8± 0.7 µ*M*, and saturates at around 30 µ*M* (Fig. 8 ▶ *b*). No inhibition was seen with ATP concentrations up to 0.5 m*M* (data not shown). To investigate the effect of metal ions and ionic strength on nick-sealing activity, Psy-Lig was assayed at a range of MgCl_2_ and NaCl concentrations. MgCl_2_ was required for Psy-Lig activity, with an optimal concentration between 1 and 5 m*M* (Fig. 8 ▶ *c*). By contrast, NaCl was not necessary for activity, but concentrations above 100 m*M* had an inhibitory effect (Fig. 8 ▶ *d*). To confirm that the decrease in activity was owing to the enzyme rather than the behaviour of the MB at high salt concentration, the NaCl assay was repeated using the gel-based endpoint assay, with the same result (Fig. 8 ▶ *d*, dashed line).

## 4. Discussion   

### 4.1. Mechanistic implications of the Psy-Lig structure for DNA binding   

The intrinsic ability of Psy-Lig to efficiently seal breaks in DNA, coupled with the absence of obvious DNA-binding domains in the crystal structure, suggests that this enzyme must have a mechanism of engaging its DNA substrate which does not rely on the ordering of large flexible loop regions in the presence of DNA, but more likely takes place through interactions with shorter highly structured motifs and specific charged residues. The most salient difference between the ChlV-Lig and Psy-Lig structures is the absence of the 30-amino-acid lysine-rich DNA-binding latch region which is disordered in ChlV-Lig–AMP and structured in the DNA-bound form (Nair *et al.*, 2007 ▶; Odell *et al.*, 2000 ▶). Point mutations in the ChlV-Lig latch diminished the nick-sealing activity, and replacement of the latch by a short linker reduced DNA binding by tenfold and increased the sensitivity to NaCl (Nair *et al.*, 2007 ▶). The Psy-Lig OB domain has a shorter loop (eight amino acids) rather than the latch, and this is fully resolved in the electron density despite the absence of DNA (Fig. 5 ▶). The results of nick-sealing assays with Psy-Lig show that it is able to efficiently seal single-stranded breaks in DNA (Figs. 7 ▶ and 8 ▶) and was partially functional at NaCl concentrations of up to 400 m*M* (Fig. 8 ▶ *d*). The loop contains a pair of lysine residues, which we postulate to be involved in DNA binding (Figs. 6 ▶ *b* and 6 ▶ *c*). The whole loop is stabilized by hydrogen bonds in the 3_10_-helical element and between the base of the loop and the main OB domain *via* Arg222. In the ChlV-Lig–DNA structure, the latch encircles the DNA duplex to form hydrogen bonds to the AD domain. In Psy-Lig the equivalent position in the AD domain, a loop between β3 and α2, is moved 2.9 Å towards the OB domain relative to the position in ChlV-Lig, and this makes the groove in which the 3′-nicked strand would lie tighter (Figs. 6 ▶ *d* and 6 ▶ *e*). The N-terminus of the Psy-Lig protein points towards the DNA-binding face under the β3–α2 loop, while in the ChlV-Lig structure it points away from the AD domain in both the DNA-bound and DNA-free structures. The other side of the groove is formed by the α3 DNA-binding helix, where Arg80 and Gln82 at the end of the helix contribute positive and polar side chains to the groove and make it deeper relative to the ChlV-Lig protein, in which the equivalent positions are Met83 and Gly85. The OB domain of T7-Lig closely resembles that of Psy-Lig in that it also has a shorter (14-amino-acid) β9–β10 loop rather than the long latch of ChlV-Lig. Instead, T7-Lig has an additional 21-amino-acid loop, in which nine residues are unstructured, that extends between the equivalent positions of α7 and β12. In Psy-Lig the α7 helix is much shorter than in either T7-Lig or ChlV-Lig, as it is broken by a stretch of three prolines. In addition to a possible contribution from the large loop in the OB domain, the T7-Lig enzyme is proposed to bind DNA *via* a 31-residue loop in the AD domain which is protease-sensitive and has no electron density for nine residues in the crystal structure (Shuman, 2009 ▶). The equivalent region in Psy-Lig is the α3–α4 stretch, which forms half of the putative DNA-binding groove but is only seven residues long and has no possibility of circling the DNA.

### 4.2. DNA ligase activity of Psy-Lig compared with other ligases   

Like the other minimal-type bacterial ADLs Nme-Lig, Hin-Lig and Vib-Lig (Cheng & Shuman, 1997 ▶; Magnet & Blanchard, 2004 ▶; Williamson & Pedersen, 2014 ▶), Psy-Lig is able to efficiently ligate singly nicked DNA when the 5′-end is phosphorylated. This is in contrast to the larger ADLs from *B. subtilis*, *Agrobacterium tumefaciens* and *M. tuberculosis*, which have poor intrinsic nick-sealing activity in the absence of the Ku partner (Weller *et al.*, 2002 ▶; Gong *et al.*, 2004 ▶; Zhu & Shuman, 2007 ▶; de Vega, 2013 ▶), although this is considerably stimulated by the presence of a single monoribonucleotide at the 3′-position of the nick (Zhu & Shuman, 2008 ▶). Nme-Lig was also able to ligate cohesive ends after extended incubation; however, no activity was observed with gapped or blunt-ended substrates (Magnet & Blanchard, 2004 ▶), while the sealing of a gapped substrate by Hin-Lig was less than 5% of its nick-sealing activity (Cheng & Shuman, 1997 ▶). The ability of Psy-Lig to act on the blunt substrate was somewhat surprising in light of the inactivity of Nme-Lig; however, it is possible that the activity of the latter was reduced by the retention of the N-terminal signal sequence in the recombinant construct tested. Comparison of the Vib-Lig homologue showed that nick sealing was increased more than twofold when the N-terminal leader sequence was removed, and it is plausible that similar truncations of Hin-Lig and Nme-Lig would stimulate their activities further. Additionally, as the temperature was not specified, it is assumed that Nme-Lig was assayed at ambient temperature. Experiments with T4 DNA ligase have shown that cohesive and blunt-ended ligation is more efficient at lower temperatures (Ferretti & Sgaramella, 1981 ▶), and this is likely to also be the case for bacterial ADLs.

The enzyme kinetics of Psy-Lig are similar to those of other small ADLs from diverse species; the affinity of Psy-Lig for DNA (*K*_m_ = 124 n*M*) is lower than that of either Nme-Lig (*K*_m_ = 30 n*M*; Magnet & Blanchard, 2004 ▶) or T4 DNA ligase (*K*_m_ = 1.5 n*M*; Hall & Lehman, 1969 ▶); however, its catalytic efficiency (*k*_cat_/*K*_m_ = 3.85 × 10^−4^) is equivalent to that of Nme-Lig (*k*_cat_/*K*_m_ = 2.83 × 10^4^) owing to the latter’s relatively slow turnover number of 0.0085 s^−1^. The *K*_m_ for ATP, 3.8 µ*M*, is similar to those of other minimal bacterial ADLs and T4 DNA ligase (Hin-Lig, 0.2 µ*M*; Nme-Lig, 0.4 µ*M*; T4 DNA ligase, 0.6 µ*M*; Cheng & Shuman, 1997 ▶; Magnet & Blanchard, 2004 ▶; Hall & Lehman, 1969 ▶) and lower than those of eukaryotes and eukaryotic viruses (mammalian ligase II, 40 µ*M*; ChlV-Ligm 75 µ*M*; *Vaccinia virus* DNA ligase, 95 µ*M*; Teraoka *et al.*, 1986 ▶; Ho *et al.*, 1997 ▶; Shuman, 1995 ▶) or the larger bacterial ADL LigB from *M. tuberculosis* (340 µ*M*; Gong *et al.*, 2004 ▶).

As with other DNA ligases, Psy-Lig absolutely requires MgCl_2_ for ligation activity. Mg^2+^ is the preferred metal for all ligases studied to date, but Mn^2+^ can also support some activity for Nme-Lig and Hin-Lig, as can Ni^2+^, Ca^2+^ and Co^2+^ to a much lesser extent (Cheng & Shuman, 1997 ▶; Magnet & Blanchard, 2004 ▶). The sensitivity of Psy-Lig to NaCl concentration was somewhat surprising, given that the predicted periplasmic location of this enzyme in a marine bacterium would expose it to NaCl concentrations of up to 600 m*M*. NaCl sensitivity of ligase activity has been reported for a number of ADLs; for example, the enzyme from *Methanocaldococcus jannaschii* shows a significant decrease above 50 m*M* (Wang *et al.*, 2013 ▶), while for T4 DNA ligase the affinity for DNA is affected, with the *K*_m_ increasing fourfold in the presence of 200 n*M* NaCl (Cherepanov & de Vries, 2003 ▶).

### 4.3. Sequence homology with other putative periplasmic ligases   

Many of the minimal ADLs from gammaproteobacteria have strong predictions of cleavable leader sequences that direct them to the periplasmic space. Previous suggestions for the biological role of such periplasmically localized DNA ligases include the protection and repair of exogenous DNA prior to uptake during transformation (Magnet & Blanchard, 2004 ▶), and although the localization of these enzymes has yet to be directly verified, many bacterial species possessing this type of ADL have been demonstrated to be naturally competent (Johnston *et al.*, 2014 ▶).

Psy-Lig has high sequence homology with other previously characterized minimal bacterial ADLs: Nme-Lig (43% identity, 71% similarity), Hin-Lig (41% identity, 76% similarity) and Vib-Lig (44% identity, 75% similarity). All four sequences are remarkably similar in length, with the greatest variation being in the N-terminal 30 amino acids where the periplasmic leader sequence is predicted. In addition to the conserved motifs found in all nucleotidyltransferase enzymes (I, III, IIIa, IV, V and VI marked in Fig. 2 ▶ *b*), areas of sequence similarity are found for most of the putative DNA-interaction sites identified in the Psy-Lig structure. The β9–β10 loop which occupies an equivalent position to the ChlV-Lig latch has a fully conserved GKG motif followed by a positive (Lys or Arg) or polar (Gln) residue and then in the case of Psy-Lig, Vib-Lig and Hin-Lig an aromatic side chain (Tyr or Phe) and the glutamate. Gly199 and Ala200 which contribute to the partial positive surface of the Psy-Lig OB domain through their backbone amides are also fully conserved, as are most of the residues from the beginning of β11 to the beginning of α7 which line the concave OB-domain surface. All three homologues have two or three prolines immediately after α7, suggesting that, as in Psy-Lig, this helix is truncated compared with the ChlV-Lig and T7-Lig structures. Conserved regions are also seen in the AD domain. In particular, the β3–α2 loop includes a number of conserved or homologous residues and all three have bulky charged side chains at the end of α3.

The sequence-based alignment of Psy-Lig with the three other biochemically characterized minimal bacterial ADLs (Fig. 2 ▶ *b*) indicates that all four homologues lack large loop regions but the majority of the putative DNA-binding residues identified in Psy-Lig are conserved. This suggests that a similar mode of DNA binding may be common to these minimal bacterial ADLs. The Vib-Lig enzyme, like Psy-Lig, was sourced from a psychrophilic marine bacterium, while Hin-Lig and Nme-Lig are from mesophilic human pathogens. Like Psy-Lig, all three homologues are competent in independent adenylation and nick-sealing activities without requiring additional interaction partners. That Psy-Lig has fewer unstructured regions and shorter loops compared with homologous structures from ChlV-Lig and T7-Lig was somewhat counterintuitive given the psychotolerant origin of Psy-Lig. However, the sequence similarity of Psy-Lig to the mesophilic Hin-Lig and Nme-Lig suggests that this cannot be attributed to psychrophilicity, but rather some common feature of this type of bacterial ATP-dependent DNA ligase. The absence of flexible loops would be consistent as an adaptation to their predicted periplasmic location as the bacterial periplasam contains a large number of proteases (Merdanovic *et al.*, 2011 ▶). Prior to structural determination, the latch region of ChlV-Lig and the AD and OB loops of T7-Lig had already been delineated as potential sites of dynamic interaction based on their protease sensitivity (Odell & Shuman, 1999 ▶; Subramanya *et al.*, 1996 ▶), therefore for a periplasmically located ligase there would be a strong selective pressure towards structures which do not require labile regions that are susceptible to degradation.

## 5. Conclusions   

In summary, we have conducted a detailed structural and biochemical study of Psy-Lig, a previously uncharacterized ATP-dependent DNA ligase. The absence of extensive loops or disordered regions in the high-resolution crystal structure of the enzyme–adenylate suggests that Psy-Lig binds to DNA with incomplete encirclement of the duplex using only the ridged, positively charged surfaces on its two domains. Extensive enzyme assays revealed that Psy-Lig has intrinsic ATP-dependent ligase activity for variety of double-stranded DNA substrates and has similar kinetic constants to other small ADLs from viruses and bacteria. Sequence homology with other putatively periplasmic, minimal-type bacterial ADLs suggests that the lack of loop regions could be an adaptation to the protease-rich environment in which these enzymes are expected to function.

## Supplementary Material

PDB reference: ATP-dependent DNA ligase, 4d05

## Figures and Tables

**Table 1:** MAD data-collection, reduction and refinement statistics for Psy-Lig (PDB entry 4d05)

	High-resolution SeMet	Peak	Inflection point	Remote
Data-collection statistics				
Beamline	BL14.1, BESSY	BL14.1, BESSY		
Space group	*C*2	*C*2		
Unit-cell parameters (, )	*a* = 178.43, *b* = 43.98, *c* = 89.63, = 105.92	*a* = 178.55, *b* = 43.92, *c* = 89.13, = 105.95		
Wavelength	0.918409	0.979771	0.979927	0.977455
Energy (keV)		12.654	12.652	12.684
*f*/*f* (observed) (e)		0.5/8.06	0.5/7.0	3.8/5.4
Total rotation range ()	300	720	360	360
Resolution ()	441.65 (1.741.65)	442.73 (2.882.73)	442.89 (3.052.89)	442.84 (3.002.84)
No. of unique reflections	80785 (11590)	17901 (2389)	15206 (2134)	16011 (2232)
*R*_merge_ (%)	7.3 (60.1)	25.3 (78.4)	20.2 (57.6)	19.3 (57.3)
Mean *I*/(*I*)	13.3 (2.1)	9.6 (3.0)	8.2 (3.0)	9.2 (3.0)
Completeness (%)	99.6 (98.8)	98.8 (91.9)	99.6 (97.2)	99.4 (96.0)
Multiplicity	4.5 (4.3)	13.2 (11.8)	6.7 (6.8)	6.7 (6.8)
MAD phasing				
Resolution ()		253.2		
No. of Se sites		13		
FOM (after *SHELXE*)		0.726 [1.65]		
Pseudo-free CC after *SHELXE* (%)		75.6 [1.65]		
Refinement				
Resolution range ()	251.65			
*R*/*R*_free_ (%)	14.54/19.24			
*B* factor (^2^)				
Protein (chains *A*/*B*)	11.6/32.8			
Water	28.0			
AMP	15.4			
Mg	41.4			
Sulfate	35.0			
R.m.s. deviation, bonds ()	0.013			
R.m.s. deviation, angles ()	1.439			
Ramachandran plot				
Favoured (%)	96.9			
Allowed (%)	3.1			
Disallowed (%)	0			

**Table 2:** Sequences of the oligonucleotides used to make ds-DNA substrates ‘Strand’ refers to the moiety that the strand contributes to the nick or to its position in the complement. (P) indicates 5 phosphorylation; other modifications are as described in the text.

Strand	Oligonucleotide sequence 5 to 3		Substrates
3-OH of nick	1	CGCACGAGA	Molecular beacon
	2	(FAM)-CGCACGAGA	Nicked, nonphosphorylated, mismatch, cohesive, blunt, gapped, single-stranded
5-P of nick	3	(P)-AGTGGAACC	Molecular beacon, nicked, mismatch, cohesive, blunt, single-stranded
	4	AGTGGAACC	Nonphosphorylated
	5	(P)-GTGGAACC	Gapped
Complement	6	(TAMRA)-CGTTGATGGTTCCACTTCTCGTGCGTTCAACG-(DABCYL)	Molecular beacon
	7	CGTTGATGGTTCCACTTCTCGTGCGTTCAACG	Nicked, gapped, nonphosphorylated
	8	CGTTGATGGTTCCACTGCTCGTGCGTTCAACG	Mismatch
3 complement	9	CGTTGATGGTTCCACT	Blunt
	10	CGTTGATGGTTCCACTTCTC	Cohesive
5 complement	11	(P)-TCTCGTGCGTTCAACG	Blunt
	12	(P)-GTGCGTTCAACG	Cohesive

**Table 3:** Oligonucleotide components annealed to make different ds-DNA substrates as described in the text The numbers correspond to those in Table 2 ▶.

Substrate	3-OH of nick	5-P of nick	Complement
Molecular beacon	1	3	6
Nicked	2	3	7
Mismatch	2	3	8
Cohesive	2	3	10 + 12
Blunt	2	3	9 + 11
Gapped	2	5	7
Nonphosphorylated	2	4	7
Single-stranded	2	3	

**Table 4:** Alignment of Psy-Lig with other ADLs of known structure Sequence homology is based on the full-length amino-acid sequences of ChlV-Lig and T7-Lig and the ligase domain of Mtu-Lig (residues 453759). The Psy-Lig signal peptide was omitted from the primary sequence alignment. Structure-based alignments of the AD and OB domains were performed. Length is the number of matched amino acids in the alignment based on three-dimensional superposition. Root-mean-square deviation (r.m.s.d.) is calculated between C atoms matched in the three-dimensional alignment. Aligned secondary-structural elements (SSEs) are the number of homologous SSEs in Psy-Lig identified in each target. The *Z*-score is the significance of the alignment based on Gaussian statistics.

		AD domain				OB domain			
	Sequence homology (*E*-value)	Length	R.m.s.d. ()	Aligned SSEs	*Z*-score	Length	R.m.s.d. ()	Aligned SSEs	*Z*-score
ChlV-LigAMP		162	2.06	10	9.0	67	0.85	4	8.0
ChlV-LigDNA	4.0 10^8^	163	2.02	11	9.7	72	0.81	4	7.2
MTu-Lig	0.26	151	2.26	9	7.8	68	1.86	5	5.1
T7-Lig	2.0 10^4^	157	1.91	10	8.5	72	1.72	5	6.7
